# Efficient identification of SNPs in pooled DNA samples using a dual mononucleotide addition-based sequencing method

**DOI:** 10.1007/s00438-017-1332-2

**Published:** 2017-06-13

**Authors:** Changchang Cao, Rongfang Pan, Jun Tan, Xiao Sun, Pengfeng Xiao

**Affiliations:** State Key Laboratory of Bioelectronics, School of Biological Science and Medical Engineering, Southeast University, Nanjing, 210096 China

**Keywords:** Dual mononucleotide addition-based sequencing, SNP identification, Pooled samples, *Epds*

## Abstract

Identifying single nucleotide polymorphism (SNPs) from pooled samples is critical for many studies and applications. SNPs determined by next-generation sequencing results may suffer from errors in both base calling and read mapping. Taking advantage of dual mononucleotide addition-based pyrosequencing, we present *Epds*, a method to efficiently identify SNPs from pooled DNA samples. On the basis of only five patterns of non-synchronistic extensions between the wild and mutant sequences using dual mononucleotide addition-based pyrosequencing, we employed an enumerative algorithm to infer the mutant locus and estimate the proportion of mutant sequence. According to the profiles resulting from three runs with distinct dual mononucleotide additions, *Epds* could recover the mutant bases. Results showed that our method had a false-positive rate of less than 3%. Series of simulations revealed that *Epds* outperformed the current method (*PSM*) in many situations. Finally, experiments based on profiles produced by real sequencing proved that our method could be successfully applied for the identification of mutants from pooled samples. The software for implementing this method and the experimental data are available at http://bioinfo.seu.edu.cn/Epds.

**Electronic supplementary material:**

The online version of this article (doi:10.1007/s00438-017-1332-2) contains supplementary material, which is available to authorized users.

## Introduction

A single nucleotide polymorphism (SNP) is a variation among individuals at a single position in a DNA sequence. Currently, SNPs are the most widely used molecular markers in many genetic studies due to their abundance and the high potential for automation (Kumar et al. 2012). For instance, SNPs occur every 1000–2000 bases when two human chromosomes are compared (Sachidanandam et al. 2001; Sherry et al. 2001).

At present, SNPs are becoming powerful tools for identifying genetic factors and have been applied in many fields (Liao and Lee 2010), including human forensics and diagnostics (Brenner and Weir 2003; Mccarthy et al. 2008), animal and crop breeding (Lagudah et al. 2009; Schennink et al. 2009), and biomolecule production improvement (Lee and Lee 2005; Snyder and Francis 2005; Nijland et al. 2007). With the continual advancements in the next-generation sequencing technology, large-scale identification, mapping, and genotyping of SNPs are now feasible for numerous species (Koboldt et al. 2013). However, SNPs determined from sequencing results may suffer from incorrect base calling and misaligned reads (Nielsen et al. 2011). In addition, prior to any application of the SNP data, the discovered SNPs must be validated to identify the true SNPs.

Taking advantage of pyrosequencing pyrogram profiles for wild and pooled samples, Lin et al. (2011) proposed a method (*PSM*) for the identification of SNPs in pooled DNA samples based on the normality test and dynamic programming algorithm, which could also be applied in SNP validation. However, this method required that the dispensing order of dNTPs should be carefully designed according to the known wild sequence to guarantee sufficient signals of non-synchronistic extensions between the wild and mutant sequences (Lin et al. 2011).

In 2014, Pu et al. (2014) proposed a real-time dual mononucleotide addition-based pyrosequencing strategy. Instead of single mononucleotide addition, Pu’s strategy relies on the cyclical addition of dual mononucleotides. Compared with the traditional real-time pyrosequencing strategy, this sequencing method could use fewer reaction cycles and obtain longer read lengths (Pu et al. 2014). At present, this kind of sequencing method has been successfully applied in SNP genotyping (Pu et al. 2016); however, the dispensation order of dual mononucleotides must also be carefully selected, which is similar to *PSM*. In addition, it is necessary to know the mutant base for the design of the dispensation order, leading to its limited application in novel SNP identification.

On the basis of Pu’s sequencing method with the cyclical addition of dual mononucleotides, we present *Epds*, a method for the identification of SNPs from pooled DNA samples. Given the pyrogram profiles of wild and pooled samples, *Epds* can identify the mutant and estimate its proportion. Simulation results revealed that *Epds* had a false-positive rate of less than 3%. Comparison with current methods revealed that *Epds* performed better in many situations. Furthermore, calculations based on profiles produced by real experiments proved that *Epds* could be successfully applied in the identification of mutants from pooled samples. We hope that our method can provide an alternative strategy for SNP discovery and validation.

## Methods

### Dual mononucleotide addition-based pyrosequencing

Pu et al. (2014) proposed a real-time dual mononucleotide addition-based pyrosequencing strategy. Instead of single mononucleotide addition, Pu’s strategy relied on adding a mixture of two different mononucleotides, A + T (AT), C + G (CG), A + G (AG), C + T (CT), A + C (AC) or G + T (GT), into the cyclical reaction each time. In theory, there are six available dual mononucleotide combinations. Because all four nucleotides (A, T, G, C) are essential for complete pyrosequencing, these six distinct dual mononucleotide combinations could form three kinds of dual mononucleotide addition: AG/CT, AC/GT and AT/CG.

**Fig. 1:**
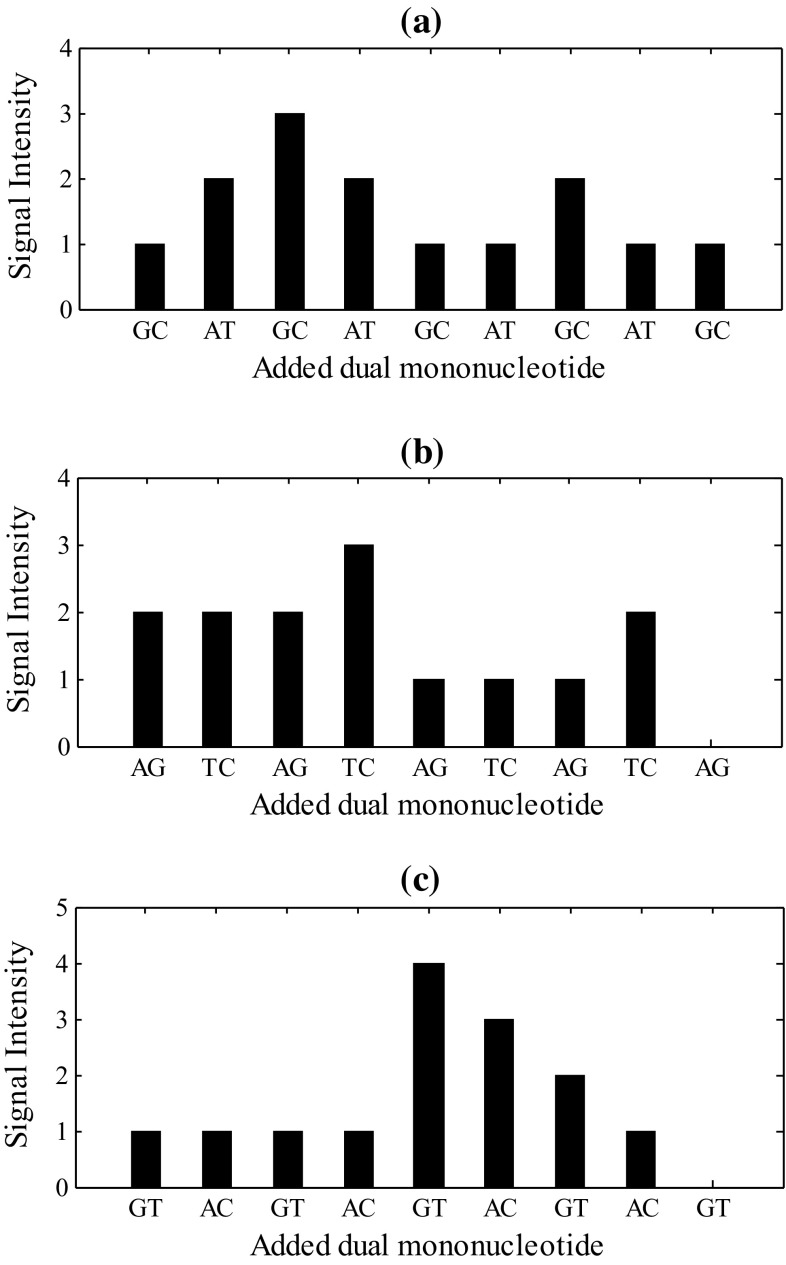
Three pyrogram profiles obtained by three pyrosequencing runs with cyclical addition of distinct dual mononucleotides for the same target sequence ‘GATCGGTTCACGTC’. The cyclical dual mononucleotide additions were **a** AT/GC, **b** AG/TC and **c** AC/GT. The dispensation orders are shown on the *x*-axis

For example, given the target sequence ‘GATCGGTTCACGTC’, utilizing three kinds of dual mononucleotide addition (AG/CT, AC/GT and AT/CG), three sets of signal intensity (also known as pyrogram profiles) could be obtained in three runs (Fig. 1). Actually, in practical experiments, the signal intensity in each reaction is proportional to, and can be translated to, the number of nucleotides extended in each reaction (Margulies et al. 2005). To describe it explicitly, we directly set signal intensity equal to the number of nucleotides extended in each reaction in the theoretical model.

On the basis of pyrogram profiles obtained by three runs with the cyclical addition of distinct dual mononucleotides, this sequencing method allows the nucleotides of the target sequence to be sequentially decoded from first to last in a deterministic manner (Pu et al. 2014). Furthermore, without sequencing errors, the recovery of the target sequence can be achieved even with only two pyrogram profiles corresponding to two sequencing runs with any two kinds of dual mononucleotide addition (Pu et al. 2014). Compared with traditional real-time sequencing strategies, this pyrosequencing strategy applies fewer cycles and can obtain longer read lengths (Ronaghi et al. 1998; Pu et al. 2014).

### Patterns of non-synchronistic extensions between the wild and mutant sequences

First, we utilize *R* (for reference) to denote the wild base and *V* (for variant) to denote the mutant base, *B* (for before) to denote the base before the mutant locus and *A* (for after) to denote the base after the mutant locus. In theory, there are five patterns of non-synchronistic extensions between the wild and mutant sequences.

**Fig. 2:**
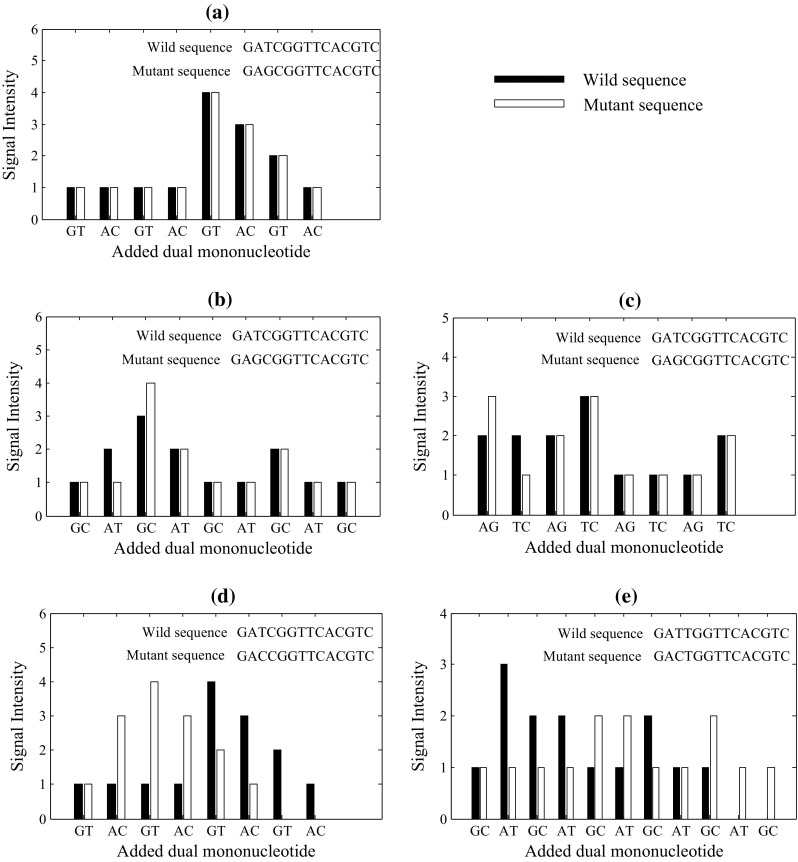
Five patterns of non-synchronistic extensions between the wild and mutant sequences: **a** Type-0, **b** Type-1, **c** Type-2, **d** Type-3 and **e** Type-4. *Black bars* represent the profiles of the wild sequence, and *white bars* represent the profiles of the mutant sequence

For a given dual mononucleotide addition, for example, AT and GC, we define two pairs of twin nucleotides: A and T, G and C. Simply, adjacent twin nucleotides can be extended in the same cyclical reaction. For a pyrosequencing run with a given cyclical addition of dual mononucleotides, if *R* and *V* are twin nucleotides, the extensions of the wild and mutant sequences will be completely synchronized. Accordingly, the profiles of the wild and mutant sequences will be identical. We define the pattern for this kind of non-synchronistic extensions as Type-0 (Fig. 2a).

When *R* and *V* are not twin nucleotides, *B* and *A* are not twin nucleotides either, and the extensions of the wild and mutant sequences will be almost synchronized during the sequencing procedure. However, the signal intensities of the wild and mutant sequences will be different in two cyclical reactions corresponding to the extensions containing *B* and *A*, respectively. Specifically, when *R* and *B* are twin nucleotides, as well as *V* and *A*, *R* and *B* will be extended in the same reaction, and *V* and *A* will be extended in the next reaction, leading to the following outcome: the signal intensity of the wild sequence will be one nucleotide higher than the signal intensity of the mutant sequence for the extension containing *B*, and the signal intensity of the wild sequence will be one nucleotide lower than the signal intensity of the mutant sequence for the extension containing *A*. Accordingly, the pattern for this kind of non-synchronistic extensions is defined as Type-1 (Fig. 2b). Conversely, when *R* and *A* are twin nucleotides, as well as *V* and *B*, the pattern is defined as Type-2 (Fig. 2c).

When *R* and *V* are not twin nucleotides but *B* and *A* are twin nucleotides, the extension of the mutant sequence will be two reactions ahead or behind the extension of the wild sequence after the mutant locus. Specifically, if *B* and *V* are twin nucleotides and *B* and *R* are not twin nucleotides, then *B*, *V* and *A* can be extended in one reaction, but *B*, *R* and *A* will be extended in three reactions. Hence, the extension of the mutant sequence will be two reactions ahead of the extension for the wild sequence after the mutant locus. Accordingly, the pattern for this kind of non-synchronistic extensions is defined as Type-3 (Fig. 2d). Conversely, when *B* and *R* are twin nucleotides and *B* and *V* are not twin nucleotides, the extension of the mutant sequence will be two reactions behind the extension of the wild sequence after the mutant locus. This pattern is defined as Type-4 (Fig. 2e). For both Type-3 and Type-4, the profiles of wild and mutant sequences may have differences in all the reactions after the mutant locus (Fig. 2d, e).

In summary, there are only five patterns of non-synchronistic extensions between the wild and mutant sequences, depending on the mutant locus and mutant base (Supplemental Fig. 1). As a result, given the profile of the wild sequence and the profile of non-synchronistic extensions, the profile of the mutant sequence could be inferred, as well as the mutant locus and mutant base.

### Estimation of the mutant proportion

Given the pyrogram profiles of wild and pooled sequences produced by dual mononucleotide addition-based pyrosequencing, we present an enumerative algorithm to estimate the proportion of the mutant and infer the mutant locus.

We use *W* = (*W*_1_, *W*_2_,…, *W*_*n*_) to denote the pyrogram profile of the wild sequence, where *W*_*i*_ stands for the signal intensity for the *i*th dispensed dual mononucleotide, and *n* denotes the time of the cyclical reaction. Likewise, we use *M* = (*M*_1_, *M*_2_,…, *M*_*n*_) to denote the profile of the mutant sequence, and *P* = (*P*_1_, *P*_2_,…, *P*_*n*_) to denote the profile of the pooled sequence. In theory,

1 $$ P = \, (1 - a)W + aM, $$

where *a* indicates the proportion of mutant sequence in the pooled samples.

Following Lin’s research (Lin et al. 2011), in real experiments, the *i*th signal intensity in profile *P* follows a normal distribution (Eq. 2):

2 $$ P_{i} \sim {\text{Normal}}(\overline{{P_{i} }} ,(\overline{{P_{i} }} \times {\text{CV}})^{2} ), $$

where $\overline{{P_{i} }}$ indicates the theoretical values, i.e., the true number of extended nucleotides for the pooled sample for the *i*th cyclical reaction. CV is the coefficient of variation, indicating the standard deviation divided by the mean; actually, CV reflects the degree of precision of the pyrosequencing experiments.

Given *W* and *P*, we define the profile of difference *D* as:

3 $$ D = P - W. $$

According to Eqs. (1) and (3), we can infer that

4 $$ D = a(M - W). $$

On the basis of Eqs. (2)–(3), we can deduce that the *i*th signal intensity in profile *D* also follows a normal distribution (Eq. 5):

5 $$ D_{i} \sim Normal(\overline{{P_{i} }} - \overline{{W_{i} }} ,(\overline{{P_{i} }} \times {\text{CV}}^{2} )), $$

where $\overline{{W_{i} }}$ indicates the true number of extended nucleotides for the pure wild sample.

Given *P* and *W*, we present an enumerative algorithm to estimate the proportion of mutant sequence and infer the mutant locus. According to the five patterns of non-synchronistic extensions, we enumerated all the possible profiles of difference between *M* and *W*, assuming that non-synchronistic extensions can begin in each cyclical reaction. Given *M*–*W*, the proportion of mutant sequence (*a*) could be estimated by Eq. (4). Because the profiles of wild and mutant sequences have different signal intensities in at least two cyclical reactions, several estimations of *a* could be obtained. Hence, the average was taken as the final estimation, denoted as $\hat{a}$.

Subsequently, we designed a score *S*_*p*_ (Eq. 6) to evaluate the possibility for each enumerated scenario:

6 $$ S_{p} = \frac{1}{n}\frac{{|D_{i} - (\overline{P}_{i} - \overline{W}_{i} )|}}{{\overline{P}_{i} {\text{CV}}}}, $$

where *n* denotes the time of the cyclical reaction. In general, *S*_*p*_ is the average of the absolute value of standard scores (also known as the *Z* score) for each element in *D*. Consequently, *S*_*p*_ could reflect the degree of deviation between the assumed and observed values for *D*. Apparently, lower *S*_*p*_ values correspond to less deviation and a higher possibility for the assumed pattern of difference between *M* and *W*.

However, because the true proportion of mutant (*a*) is unknown, $\overline{{P_{i} }}$ is also unknown. According to Eqs. (3) and (4), given the assumed profile of difference between *M* and *W*, we use $\overline{Wi} - \hat{a}(\overline{Wi} - \overline{M} i)$ to approximate $\overline{{P_{i} }}$. In addition, CV is assumed to be an unknown constant. Therefore, we modify *S*_*p*_ as Eq. (7) which neglects CV but could still reflect the degree of deviation between the assumed and observed profiles of difference:

7 $$ S_{p} = \frac{1}{n}\sum\limits_{i = 1}^{n} {\frac{|{D_{i} - \hat{a}(\overline{{M_{i} }} - \overline{{W_{i} }} )}|}{{\overline{{W_{i}}} - \hat{a}({\overline{{W_{i} }} - \overline{{M_{i} }} )}} }} . $$

In summary, given the profiles for wild sequence (*W*) and pooled samples (*P*), on the basis of five distinct patterns, we enumerated all the scenarios in which non-synchronistic extensions begin in each cyclical reaction and obtained the difference between the *M* and *W*, followed by an estimation of the proportion of mutant sequence using Eq. (4). Simultaneously, we also calculated the score *S*_*p*_ to evaluate the possibility for each assumed scenario. Finally, we chose the assumed scenario which could minimize *S*_*p*_.

### Identification of mutant locus and base

Given the difference between *M* and *W*, the mutant locus could be identified as the locus where non-synchronistic extension begins. We assumed that non-synchronistic extension begins in the *j*th cyclical reaction.

Obviously, the number of synchronistically extended nucleotides before the *j*th reaction could be counted accurately and directly according to the pyrogram profile of the wild sequence. The number of synchronistically extended nucleotides in the *j*th reaction, denoted as *S*, is the minimum number of nucleotides between $\overline{M_j}$ and $\overline{W_j}$. In theory, *S* is equal to $\overline{M_j}$ for Type-1 and Type-4, and is equal to $\overline{W_j}$ for Type-2 and Type-3 (Fig. 2).

For Type-1, $\overline{M_j}$ is equal to $\overline{W_j} - 1$. For Type-4, according to Eq. (1), $\overline{M_j}$ can be determined by Eq. (8):

8 $$ \overline{{M_{j} }} = \overline{{W_{j} }} + \frac{{\overline{{P_{j} }} - \overline{{W_{j} }} }}{a}. $$

In summary, we can formulate *S* as in Eq. (9). The predicted mutant locus *L* can be formulated as inEq. (10).

9 $$ S = \left\{ {\begin{array}{*{20}l} {\overline{{W_{j} }} - 1} \\ {\overline{{W_{j} }} } \\ {\overline{{W_{j} }} + (P_{j} - \overline{{W_{j} }} )/\hat{a}} \\ \end{array} } \right.\begin{array}{*{20}l} \quad {\text{for Type-1}} \\ \quad {\text{for Type-2 and Type-3}} \\ \quad {\text{for Type-4}} \\ \end{array} , $$

10 $$ L = \sum\limits_{i = 1}^{j - 1} {\overline{{W_{i} }} + S + 1} , $$

where $\hat{a}$ is used to approximate *a*, and $P_j - \overline{W}_j$ is used to approximate $\overline{P}_{j}  - \overline{W}_{j}$.

At the same time, we could also read the possible nucleotides for mutant base. For Type-2 and Type-3 patterns of difference, the set of possible nucleotides for mutant bases is identical to the set of nucleotides added in the *j*th reaction. Conversely, for Type-1 and Type-4 patterns of difference, the set of possible nucleotides for mutant bases is identical to the set of nucleotides added in the *j* + 1th reaction.

### Mutant recovery

Since in one of three runs, the wild and mutant bases will be twin nucleotides, i.e., the profiles of wild and mutant sequences will be identical, it leads to identical profiles of wild sequence and pooled samples Therefore, given pyrogram profiles for three runs, we first need to identify in which run the wild and mutant bases are twin nucleotides.

Similar to Eq. (7), we design a score *S*_*c*_ (Eq. (11)) to evaluate the degree of consistency between the profiles of wild sequence and pooled sample:

11 $$ S_{c} = \frac{1}{n}\sum\limits_{i = 1}^{n} {\frac{{|D_{i} |}}{{P_{i} }}} , $$

where *n* denotes the time of the cyclical reaction. Actually, *S*_*c*_ is a special case of *S*_*p*_ (Eq. 7) in which *M* and *W* are supposed to be identical. Obviously, *S*_*c*_ could reflect the degree of deviation between *D* and zero, since the mutant is supposed to result in identical profiles for wild and mutant sequences. Considering that *S*_*p*_ reflects the degree of deviation between *D* and the assumed profiles of difference in which the mutant is supposed to result in different profiles for wild and mutant sequences, we define *S*_*m*_ (Eq. 12) to evaluate the possibility that the mutant leads to different profiles for wild and mutant sequences:

12 $$ S_{m} = 1 - \frac{{S_{p} }}{{S_{c} }}. $$

For a given kind of dual mononucleotide addition, when the mutant leads to different profiles for wild and mutant sequences, *S*_*c*_ will be greater than *S*_*p*_. *S*_*m*_ will be greater for a higher proportion of the mutant sequence. Intuitively, *S*_*m*_ reflects the impact of the mutant on the consistency between the profiles of the wild sequence and pooled samples.

We can infer that the run with a smaller *S*_*m*_ than the other two runs will produce identical profiles for the wild sequence and pooled samples. When the mutant loci inferred from the other two runs are identical, the mutant locus can be determined directly, and the proportion of the mutant can be calculated as the average of the two estimated proportions. Finally, according to the set of possible nucleotides for mutant base, we can call the consensus base, i.e., the base supported by all the base sets.

Due to the random noise within the signal intensity, our algorithm will obtain a presumed mutant for each run with a given dual mononucleotide addition whether mutants exist in the pooled sample or not. When the mutant loci inferred from the specified two runs are inconsistent, we will assert that no mutant sequence exists.

**Fig. 3:**
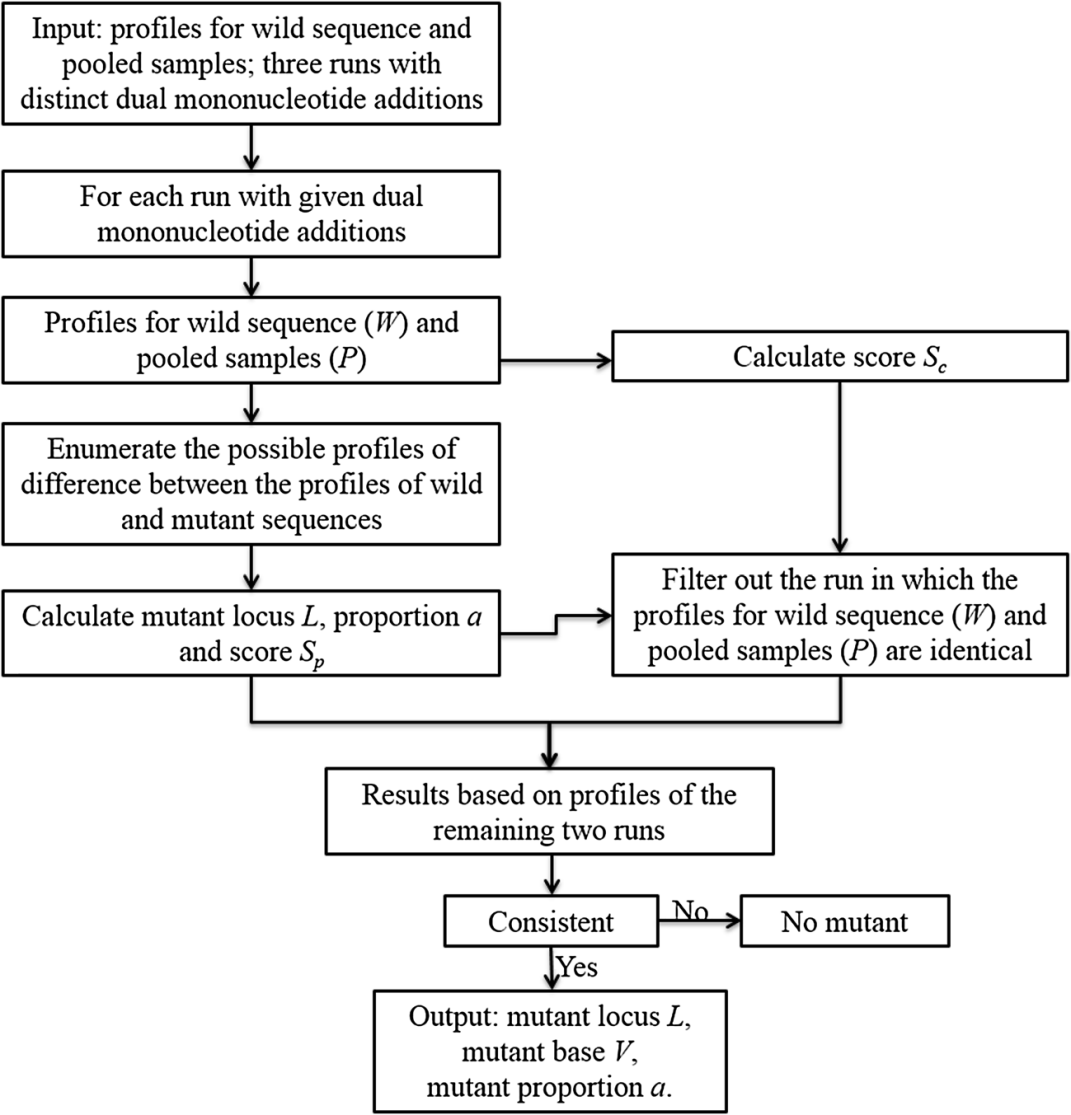
The framework of *Epds*

All of the procedures used in our method are shown in Fig. 3. In summary, using three pyrogram profiles for both wild and pooled samples produced by three pyrosequencing runs with distinct dual mononucleotide additions, our method can predict the locus, base and proportion of the mutant. A simple example is also provided to illustrate the details of *Epds* (Supplemental Appendix 1).

### Detection of single nucleotide insertion or deletion

Similar to single nucleotide substitution, two possible patterns of non-synchronistic extensions (denoted as Type-i-1 and Type-i-2) caused by a single nucleotide insertion and two possible patterns of non-synchronistic extensions (denoted as Type-d-1 and Type-d-2) caused by a single nucleotide deletion are also summarized in Supplemental Fig. 2.

Based on these patterns of non-synchronistic extensions between wild and mutant sequences with single nucleotide insertions or deletions, we also presented *Epds*-*ins* and *Epds*-*del* to detect single nucleotide insertions and deletions using the similar enumerate algorithm in *Epds*. Because insertions and deletions lead to distinct profiles of wild and mutant sequences for any kind of dual mononucleotide addition, *Epds*-*ins* (*Epds*-*del*) asserts an insertion (deletion) in the pooled sample only when the mutant loci inferred from the three runs are consistent.

## Results

### Performance of *Epds*

First, we conducted a series of simulations to investigate the performance of *Epds*. Random wild sequences of fixed lengths of 50 bp were simulated. Next, we set the mutant locus and mutant base randomly to construct the mutant sequence and used Perl scripts to produce artificial pyrogram profiles for both wild and pooled sequences, where the mutant proportions ranged from 0.01 to 0.64, and the CV for the signal intensity ranged from 0.0001 to 0.2048. Utilizing *Epds*, we predicted the mutant locus, the mutant proportion and the mutant bases for each replicate. For each scenario, 10,000 replicates were conducted. For a given replicate, we regarded it as correctly calculated only when the predicted mutant locus and mutant base were identical to the inputs.

**Fig. 4:**
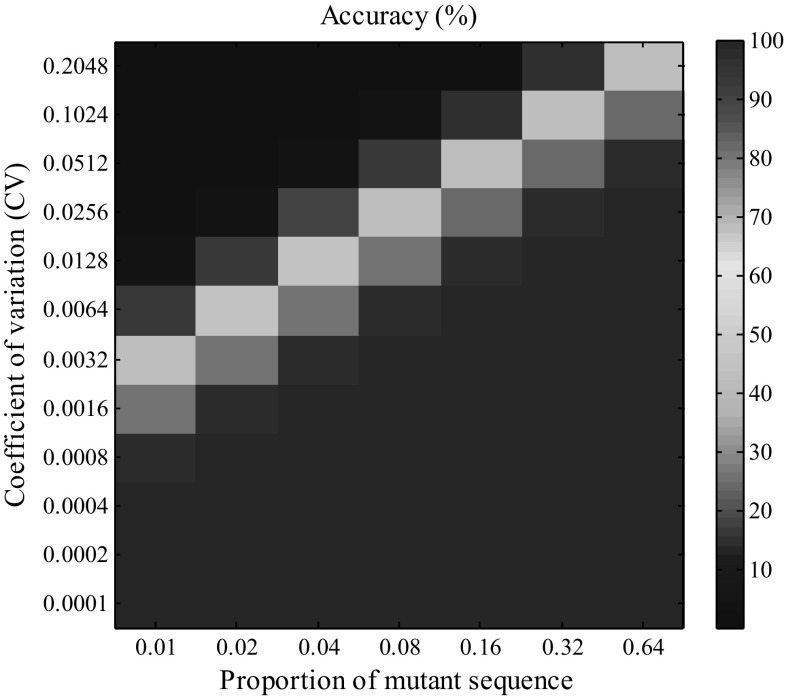
The accuracy of our method for various proportions of mutant sequence and coefficients of variation (CV). Ten thousand replicates were conducted for each scenario

We used the percentage of correctly calculated replicates to evaluate the accuracy of our method (Fig. 4; Supplemental Fig. 3). As an example, our method achieved an accuracy greater than 98% for mutants with proportions higher than 0.02 in the pooled samples when the value of CV was fixed as 0.0016; however, the accuracy dropped to 44% when the CV increased to 0.0064. These results revealed that when the value of CV is lower or the mutant proportion higher, our method can achieve greater accuracy, as both higher CV and lower mutant proportion will make it harder to separate the profiles of true difference from the random background noise and hinder the correct calculation.

**Fig. 5:**
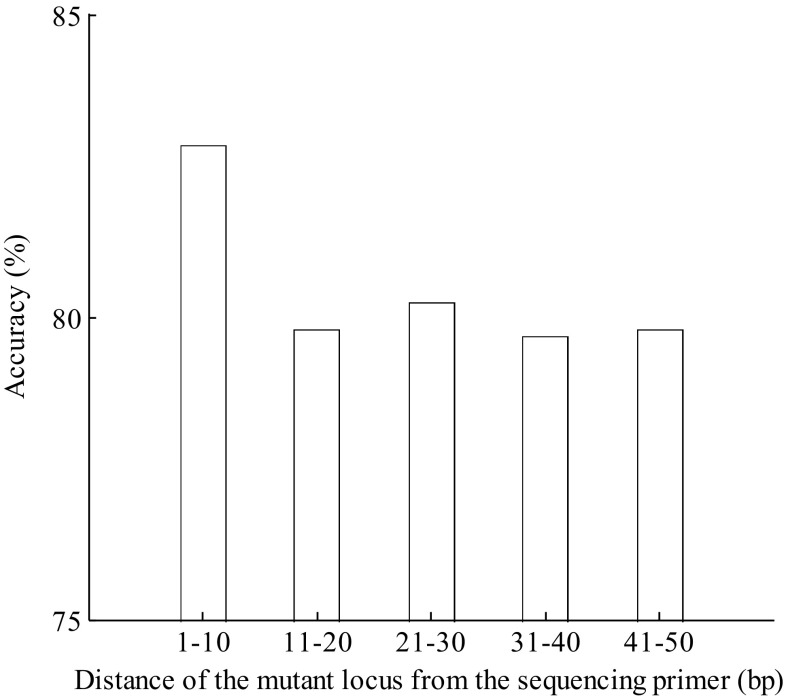
The accuracy of *Epds* is related to the distance of the mutant locus from the sequencing primer

Next, we analyzed the correlation between the accuracy of our method and the mutant loci when the CV was fixed at 0.0064 and the mutant proportion was fixed at 0.04. Under this condition, our method achieved an average accuracy of 80%. The results showed that the accuracy decreased with the distance of the mutant locus from the sequencing primer (Fig. 5). When the mutant locus is located close to the sequencing primer, signals resulting from non-synchronistic extensions will be more significant and can be more easily distinguished from the background noise. Therefore, our method was more accurate for mutants located closer to the sequencing primer.

**Fig. 6:**
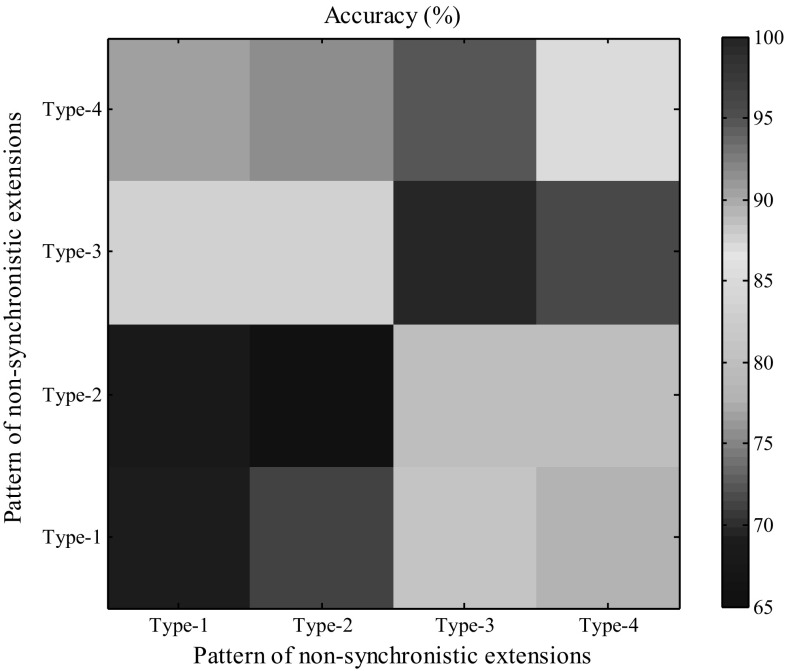
Impact of the patterns of non-synchronistic extensions on the accuracy of our method

According to previous analyses, there are five patterns of non-synchronistic extensions between the wild and mutant sequences. In one pyrosequencing run, the pattern must be Type-0, where the profiles for wild and mutant sequences are identical. Therefore, we only analyzed the association between the accuracy of our method and the other two patterns corresponding to the other two runs. We still fixed the CV at 0.0064 and the mutant proportion at 0.04. The results revealed that our method had higher accuracies for Type-3 and Type-4 and lower accuracies for Type-1 and Type-2 (Fig. 6). When both patterns were Type-3, the accuracy reached 99.7%. Because the profile of difference (*D*) for Type-1 and Type-2 patterns only have two non-zero elements, it is hard to distinguish the real difference from the background noise. However, *D* may have numerous non-zero elements after the mutant locus for Type-3 and Type-4.

In summary, we can infer that when the signals resulting from non-synchronistic extensions are more significant, for example when the pattern is Type-3 or Type-4 or the mutant locus is closer to the sequencing primer, our method will achieve higher accuracy.

We also simulated the scenario in which no mutant sequence existed in the pooled samples. For a given replicate, it was regarded as correctly calculated only when our method asserted that no mutant sequence existed. With different values of CV, we conducted 10,000 replicates for each scenario and counted the percentage of correctly calculated replicates. The results indicated that our method could attain an accuracy greater than 97% (Supplemental Fig. 4), and the estimated proportion of falsely predicted mutants is related to the value of CV (Supplemental Fig. 5). Since we assert a mutant only when the mutant loci inferred from two runs are identical, our method showed robustness to CV when no mutant sequence existed.

### Comparison with *PSM*

In 2011, Lin et al. (2011) presented a pyrosequencing-based method for the identification of SNPs in pooled DNA samples and provided a Web service (denoted as *PSM*, http://life.nctu.edu.tw/~yslin/PSM/). In this research, Lin et al. conducted simulation experiments to investigate the performance of *PSM* using four pairs of wild and mutant sequences (Supplemental Table 1). Because *PSM* is not available for large-scale simulations, we utilized the same sequences to compare the accuracy of our method with the accuracy of *PSM* presented in Liu’s article (Lin et al. 2011).

**Fig. 7:**
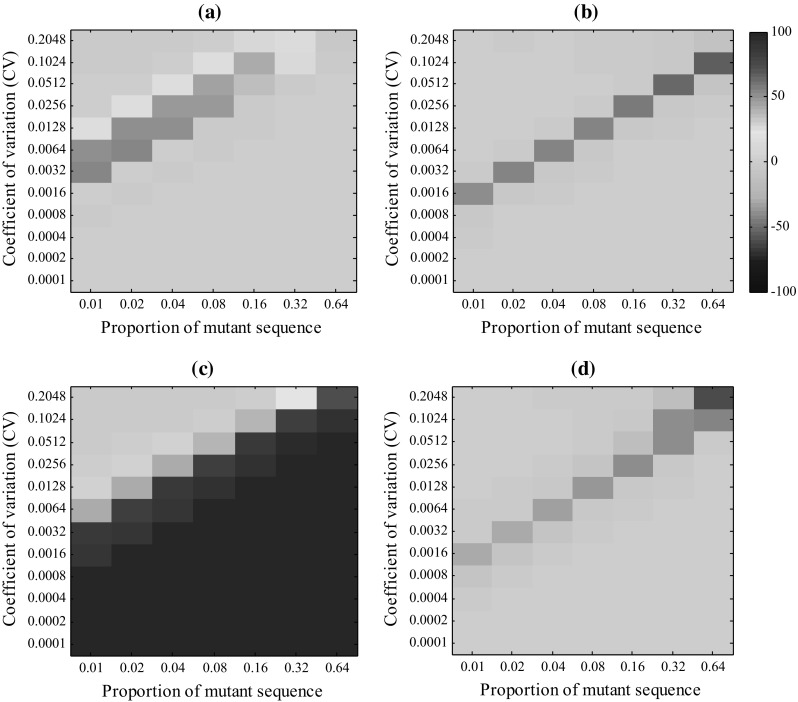
Comparison of the accuracies of our method and *PSM*, where the wild and mutant sequences are **a** Pair I, **b** Pair II, **c** Pair III and **d** Pair IV (*Epds*-*ins* was used). The data shown are the accuracies of our method minus the accuracies of *PSM*. *Warm colors* indicate that our method is more accurate, and *cool colors* indicate that *PSM* is more accurate (color figure online)

With the same procedure previously described, we counted the percentage of correctly calculated replicates for our method and compared it with the correctly calculated replicates for *PSM* (Fig. 7, detailed accuracies are listed in Supplemental File 2). The results revealed that our method performed better for Pair I and Pair III; however, *PSM* was better for Pair II and Pair IV.

For Pair I, where the mutant occurred at the third nucleotide, the profile of difference had 23 non-zero elements when normal pyrogram sequencing was used. In our method, the patterns of non-synchronistic extensions are Type-3 and Type-4. For Pair II, the mutant occurred at the 37th nucleotide, and the profile of difference had five non-zero elements when normal pyrogram sequencing was used. In our method, the patterns of non-synchronistic extensions are Type-1 and Type-2. For Pair III, the mutant occurred at the 40th nucleotide, and the profile of difference had two non-zero elements when normal pyrogram sequencing was used. In our method, the patterns of non-synchronistic extensions are Type-3 and Type-4. For Pair IV, where a guanine was inserted between the third and fourth nucleotide, the profile of difference had 22 non-zero elements when normal pyrogram sequencing was used. In our method, the three patterns of non-synchronistic extensions are Type-i-1, Type-i-1 and Type-i-2 (Supplemental Fig. 2).

In theory, our method performed better for Type-3 and Type-4 than Type-1 and Type-2 profiles; *PSM* performed better when the profile of difference contained more non-zero elements (Lin et al. 2011). According to the comparison, we can conclude that which method performed better depends greatly on the signals from non-synchronistic extensions between the wild and mutant sequences.

**Fig. 8:**
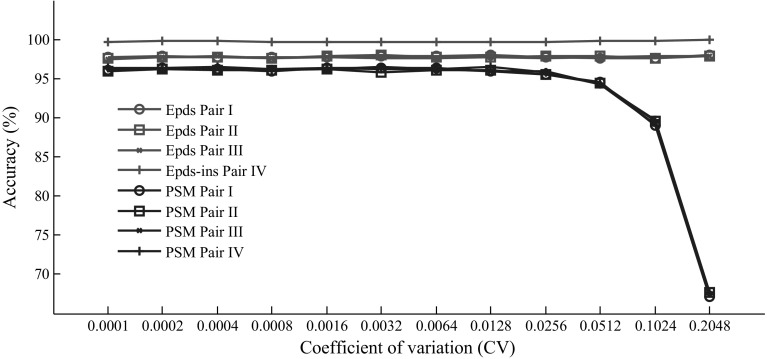
Comparison between the accuracy of our method and *PSM* when no mutant sequence exists in the pooled samples

Utilizing the same four pairs of wild and mutant sequences, we also compared the accuracy of our method with *PSM* when there was no mutant in the pooled sample (Fig. 8). The results showed that our method maintained excellent performance at high CVs, which may lead to false positive predictions of mutant sequence when using the *PSM* algorithm. *Epds*-*ins* only asserts that there is an insertion when the mutant loci inferred from the three runs were consistent; therefore, the accuracy of *Epds*-*ins* was slightly higher than *Epds* when no mutant existed in the pooled sample. This simulation also proved that in our method, three runs with distinct dual mononucleotide additions could help to reduce the false-positive prediction of mutant sequence and make *Epds* robust to CV when no mutant sequence exists.

**Fig. 9:**
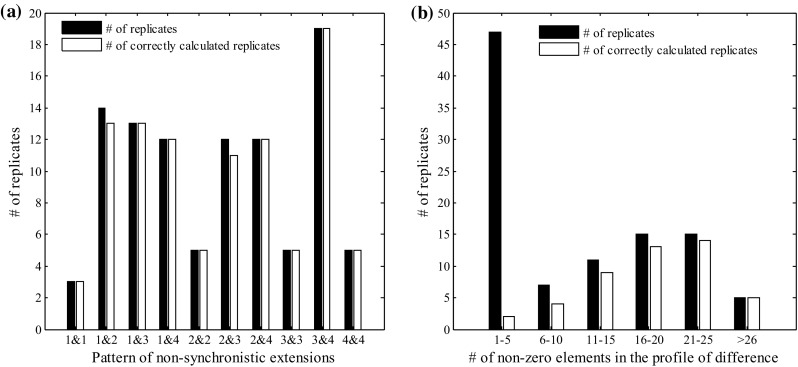
The association between the accuracy and the profiles of difference for **a** our method and **b** *PSM*. In total, 100 pairs of wild and mutant sequences were used in this analysis

Taking advantage of the Web service, we also compared the performance of our method and *PSM* for 100 pairs of randomly simulated wild and mutant sequences with fixed lengths of 40 bp where the mutant base and locus were randomly selected (sequences are provided in Supplemental File 3). In this comparison, we set the CV at 0.0064 to produce pyrogram profiles for wild and pooled samples, where the proportion of mutant sequence was fixed at 0.08. The results showed that our method achieved a much higher accuracy than *PSM* (98 vs. 47%). Furthermore, the mutant proportion estimated by our method was also more accurate (0.08008 vs. 0.0789; Supplemental Table 2; Fig. 6). As previously described, sufficient signals from non-synchronistic extensions are crucial for both our method and *PSM*. Accordingly, we also analyzed the association between the accuracy and the profiles of difference for our method and *PSM* (Fig. 9).

When using normal pyrosequencing technology, approximately half of the profiles of difference have less than five non-zero elements. This situation was the main reason that led to the accuracy of 47% for *PSM* for these 100 pairs of wild and mutant sequences. In theory, using normal pyrosequencing technology, about 40% replicates have only two non-zero elements, as long as the mutant base is identical to the base before or after the wild base (Supplemental Fig. 7). In this kind of scenario, *PSM* will reject the existence of the mutant in the pooled sample. Furthermore, *PSM* requires at least three non-zero elements in the profile of difference to estimate the proportion of mutant sequence and predict the profile of the mutant sequence (Lin et al. 2011). In our method, the pattern of non-synchronistic extensions has an equal chance of being Type-1, Type-2, Type-3 or Type-4 for a random mutant (Supplemental Fig. 7). However, *Epds* rejects the existence of a mutant in the pooled sample only when the mutant loci inferred from two runs are inconsistent, and *Epds* can use as few as two non-zero elements in the profile of difference to estimate the proportion of the mutant sequence (e.g., when the pattern is Type-1 or Type-2). Therefore, *Epds* performs better than *PSM* for random mutants.

For the *PSM* algorithm, the order of dNTP dispensation should be carefully designed according to the known wild sequence to guarantee a significant profile of difference (Lin et al. 2011). However, in our method, the dual mononucleotide is cyclically added, and the order of dispensation is identical to that used in the sequencing protocol, which could be implemented more easily.

### Verification

To further test the performance of our method, we conducted a real experiment to independently identify two SNPs from pooled samples. SNP1 (rs4986850) was located in the *BRCA1* gene and may confer increased risk for breast cancer (Johnson et al. 2007; Bhatti et al. 2008; De et al. 2009). SNP2 (rs11564148) was located in the *LRRK2* gene, which is related to Parkinson’s disease (Wu et al. 2013). The wild and mutant sequences are listed in Supplemental Table 3.

Genomic DNA was first extracted from whole human blood, and fragments containing the target SNPs were amplified by PCR. Because homozygous individuals carrying mutant-type alleles were not available for our experiment, we constructed plasmids containing the wild or mutant allele for SNP1 and SNP2 and mixed these plasmids to obtain pooled DNA samples with different proportions of mutant sequence. In this experiment, we constructed four pooled DNA samples; the proportions of mutant sequence were 0% (i.e., pure wild sequence), 3, 5 and 7%. After PCR amplification, for each sample, we replicated three times for each kind of dual mononucleotide addition-based sequencing and obtained 27 sets of profiles by freely combining profiles for three kinds of dual mononucleotide addition. Details regarding sample preparation and dual mononucleotide addition-based sequencing can be found in Supplemental Appendix 2.

Based on the profiles of the wild sequences for these two SNPs, we estimated that the coefficient of variation for the dual mononucleotide addition-based sequencing ranged from 0.0064 to 0.0128. Under this condition, we first conducted a series of simulations to predict the accuracy of *Epds* for identification of these two SNPs from pooled samples (Supplemental Fig. 8).

Before using *Epds*, the theoretical profile of wild sample for each kind of dual mononucleotide was inferred as the average values of three replicates. Subsequently, *Epds* was applied to infer the mutant and estimate its proportion based on the profiles of pooled samples and the theoretical profile of the wild sample. The accuracy of *Epds* and the estimated mutant proportion are listed in Table 1.

**Table 1:** The verification of our method based on real experiments with dual mononucleotide addition-based sequencing

Mutant proportion	0%	3%	5%	7%
SNP1				
Predicted accuracy^a^	97%	22–58%	46–91%	69–99%
Accuracy^b^	100%	52%	67%	100%
Estimated mutant proportion	NA	0.033	0.047	0.061
SNP2				
Predicted accuracy	91%	21–55%	44–85%	65–96%
Accuracy	89%	19%	33%	67%
Estimated mutant proportion	0.012	0.023	0.030	0.038

^a^Percent of correctly calculated replicates in 10,000 replicated simulations

^b^Percent of correctly calculated replicates in 27 replicates

For SNP2, the accuracy was slightly lower than the predicted accuracy, especially for the pooled sample with 5% mutant sequence. We inferred that mixing bias should be responsible for the lower accuracy, as the estimated mutant proportion was much lower than the designed proportion. Nevertheless, these results showed that our method could be successfully applied in the identification of SNPs from pooled samples.

## Discussion

In this study, by means of dual mononucleotide addition-based sequencing technique, we presented *Epds*, a method to identify SNPs from pooled DNA samples. Based on only five patterns of non-synchronistic extensions between wild and mutant sequences when dual mononucleotide addition-based sequencing was used, we proposed an enumerative algorithm to infer the mutant locus and estimate the proportion of mutant sequence. According to the profiles produced by three runs with distinct dual mononucleotide additions, *Epds* could recover the mutant bases. Large numbers of simulations revealed that the precision of the pyrogram signals and the proportions of mutant sequence in the pooled sample had a large impact on the accuracy of our method. Comparison with the current method revealed that *Epds* performed better than *PSM* in many situations, and *Epds* could be implemented more easily than *PSM* since the dual mononucleotide is cyclically added. Finally, calculation based on profiles produced by real sequencing experiments proved that our method could be successfully applied for the identification of mutants from pooled samples.

According to the patterns of non-synchronistic extensions between wild and mutant sequences with single nucleotide insertions or deletions, we provided *Epds*-*ins* and *Epds*-*del* to detect single nucleotide indels. However, users need to know the mutant types (substitutions, insertion or deletion) in advance so that a correct program could be chosen and applied. This situation limits the major applications of our method in the validation of known mutants. In future, more effort will be paid to the auto recognition of mutant types, although one temporary possible solution is to compare the score (Eq. 12) given by each program and choose the most likely mutant.

Because we assert that a mutant sequence exists only when the mutant loci inferred from two runs are identical, our method showed robustness to CV when no mutant sequence existed. However, everything is a double-edged sword. The false-positive rate of our method reached 3% when identifying an SNP from pooled samples. Whether or not mutants exist in the pooled sample, our algorithm will obtain a presumed mutant for each run with a given dual mononucleotide addition. This strategy is likely to be the source of the majority of false-positive identifications of mutants. Referring to Lin’s strategy (Lin et al. 2011), we believe that statistical tests could also be integrated in our method to calculate the degree of consistency between the profiles of wild and pooled samples and to evaluate the probability of the existence of mutants in the pooled samples. Hence, the false-positive identification rate may be reduced.

Simulation results revealed that the performance of our method depends heavily on the precision of the pyrogram signals and the proportion of mutant sequence. At present, *Epds* shows great performance for mutants of high proportion in the pooled samples. Recent research has suggested that the standard deviation of pyrosequencing signals range from 0.006 to 0.031 (Dupont et al. 2004; Ogino et al. 2005), but could be further reduced to 0.0003–0.0018 (Doostzadeh et al. 2008). Under this condition, our method can identify rare mutant alleles from pooled samples. COLD-PCR for the rapid amplification and robust enrichment of low-abundance DNA mutations will further broaden the application of our method in the identification of rare mutant alleles from pooled samples (Li et al. 2008; Coren et al. 2011). Furthermore, by taking advantage of combinatorial pooled sequencing (Cao and Sun 2016), our method may be feasible for use in screening for rare variant carriers, especially when the number of individuals are limited in the pool to guarantee enough proportion of mutant sequence.

In summary, our method shows great potential for identifying mutants from pooled samples. Given the mutant locus and mutant base, our method is more accurate for SNP validation, as long as the sign of SNPs can be observed in at least one run. One major application of our method is to identify SNPs or validate candidate SNPs for a given individual or population (Bundock et al. 2009; Kumar et al. 2012). Because SNPs discovered by current sequencing techniques are often affected by sequencing errors and misaligned reads (Kumar et al. 2012), our method could provide an alternative strategy for SNP discovery and validation.

The software for implementing the *Epds* method is open source and available for download and can be easily integrated into existing analysis pipelines.

## Electronic supplementary material

Below is the link to the electronic supplementary material.

Supplementary material 1 (DOCX 491 kb)

Supplementary material 2 (XLSX 22 kb)

Supplementary material 3 (TXT 17 kb)
