# Supplementary material for: Efficient identification of SNPs in pooled DNA samples using a dual mononucleotide addition-based sequencing method
Source: Mol Genet Genomics. 2017 Jun 13;292(5):1069–81. doi: 10.1007/s00438-017-1332-2 (PMC5594057; doi:10.1007/s00438-017-1332-2)
Supplement: Supplementary file 1 — Supplementary material 1 (DOCX 491 kb) [file 438_2017_1332_MOESM1_ESM.docx]

Efficient identification of SNPs in pooled DNA samples using a dual mononucleotide addition-based sequencing method

Changchang Cao · Rongfang Pan · Jun Tan · Xiao Sun^*^· Pengfeng Xiao^*^

State Key Laboratory of Bioelectronics, School of Biological Science and Medical Engineering, Southeast University, Nanjing 210096, China

Changchang Cao and Rongfang Pan contributed equally to this work.

^*^ To whom correspondence should be addressed. Tel: +86 25 83795174; Email: xsun@seu.edu.cn (Xiao Sun) and xiaopf@seu.edu.cn (Pengfeng Xiao).

**Fig. 1** Five patterns of non-synchronistic extensions between the wild and mutant sequences. In this figure, each column denotes a pattern and six rows in each column indicates whether the corresponding nucleotides are twin-nucleotides or not. Black block denotes that the nucleotides are twin-nucleotides, white block denotes that the nucleotides are not twin-nucleotides, gray block denotes both scenarios. *R* (for reference) stands for wild base and *V* (for variant) for mutant base, *B* (for before) for the base before the mutant locus and *A* (for after) for the base after the mutant locus.

**Fig. 2** **Up panel**: Two patterns of non-synchronistic extensions between the wild and mutant sequences with single nucleotide insertion. (a) Type-i-1: the extension for mutant sequence will be two reactions behind the extension for wild sequence after the mutant locus. (b) Type-i-2: The signal intensity of wild sequence will be one nucleotide lower than the signal intensity of mutant sequence for the extension containing the insertion. **Down panel**: Two patterns of non-synchronistic extensions between the wild and mutant sequences with single nucleotide deletion. (c) Type-d-1: the extension for mutant sequence will be two reactions ahead the extension for wild sequence after the deletion. (d) Type-d-2: The signal intensity of wild sequence will be one nucleotide higher than the signal intensity of mutant sequence for the extension containing the deletion.

**Fig. 3** The accuracy of *Epds* for various proportions of mutant sequence and coefficients of variation (*CV*). Different proportions of mutant sequence were denoted by distinct colors and marks.

**Fig. 4** The accuracy of *Epds* when no mutant sequence exists in the pooled samples.

**Fig. 5** The estimated proportion for the false predicted mutant when no mutant sequence exists in the pooled samples

**Fig. 6** The estimated proportion of mutant sequence when *Epds* (our method) and *PSM* were utilized respectively.

**Fig. 7** The profiles of difference for 100000 pairs of randomly simulated wild and mutant sequences with length fixed as 40bp. (a) our method. (b) *PSM*.

**Fig. 8** The predicted accuracy of our method for the verification experiment.

**Table 1** Four pairs of wild and mutant sequences used in Lin’s research

| Pair I | Mutant locus | 3 |
| --- | --- | --- |
|  | Wild seq | ACACCAAGTCGTGTTCACAGTGGCTAAGTTCCGCCAGCCTCAC |
|  | Mutant seq | ACGCCAAGTCGTGTTCACAGTGGCTAAGTTCCGCCAGCCTCAC |
| Pair II | Mutant locus | 37 |
|  | Wild seq | ACACCAAGTCGTGTTCACAGTGGCTAAGTTCCGCCAGCCTCAC |
|  | Mutant seq | ACACCAAGTCGTGTTCACAGTGGCTAAGTTCCGCCATCCTCAC |
| Pair III | Mutant locus | 40 |
|  | Wild seq | ACACCAAGTCGTGTTCACAGTGGCTAAGTTCCGCCAGCCTCAC |
|  | Mutant seq | ACACCAAGTCGTGTTCACAGTGGCTAAGTTCCGCCAGCCACAC |
| Pair IV | Insertion locus | 3 |
|  | Wild seq | ACA-CCAAGTCGTGTTCACAGTGGCTAAGTTCCGCCAGCCTCAC |
|  | Mutant seq | ACAGCCAAGTCGTGTTCACAGTGGCTAAGTTCCGCCAGCCTCAC |

**Table 2** Comparison between the accuracy of our method and *PSM* for 100 pairs of randomly simulated wild and mutant sequences with length fixed as 40bp where mutant base and locus were randomly selected

| Method | *Epds* | *PSM* |
| --- | --- | --- |
| Accuracy | 98% | 47% |
| Average Estimated proportion | 0.08008 | 0.0789 |

**Table 3** The wild and mutant sequences used in the real experiment

| SNP1 | Wild sequence | Chr17, 43093434-43093474,  CTCTGGGAAAGTATCGCTGTCATGTCTTTTACTTGTCTGTT |
| --- | --- | --- |
|  | Mutant sequence | Chr17, 43093434-43093474,  CTCTGGGAAAGTATCGCTGTTATGTCTTTTACTTGTCTGTT |
|  | Pattern of non-synchronistic extensions | Type-2 and Type-3 |
| SNP2 | Wild sequence | Chr12, 40713895-40713906  TACATGTCACAG |
|  | Mutant sequence | Chr12, 40713895-40713906  TACATGACACAG |
|  | Pattern of non-synchronistic extensions | Type-1 and Type-2 |

**Appendix 1** A simple example to illustrate our method

In this case, the wild sequence is ‘GATCGGTTCACGTC’, the mutant sequence is ‘GACCGGTTCACGTC’. Pooled sample consists of 95% wild and 5% mutant sequences. Mutant happens at the 3rd nucleotide. The wild base (*R*) is T, mutant base (*V*) is C, the base before the mutant locus (*B*) is A, the base after the mutant locus (*A*) is C.

1. The patterns of non-synchronistic extensions between the wild and mutant sequences (Fig. 1).

Fig.1 Difference between the profiles of wild and mutant sequence for three runs with three kinds of dual mononucleotide combinations. (a) The cyclically added two dual mononucleotide combinations are AT and GC, the pattern for the non-synchronistic extensions is Type-1. (b) The cyclically added two dual mononucleotide combinations are AG and TC, the pattern for the non-synchronistic extensions is Type-0. (c) The cyclically added two dual mononucleotide combinations are AC and GT, the pattern for the non-synchronistic extensions is Type-3.

2. Difference between the profiles of wild and pooled sequences. Pooled sample consists of 95% wild and 5% mutant sequences. Fix the cyclical dual mononucleotide as AT and GC, the pattern of non-synchronistic extensions between the profiles of wild and mutant sequences is Type-1 (Fig. 2).

Fig.2 (a) The profiles for wild and mutant sequences. (b) Difference between the profiles for wild and mutant sequences. (c) The profiles for wild and pooled sequences. (d). Difference between the profiles for wild and pooled sequences.

3. Given the profiles of wild and pooled sequences, mutant proportion could be estimated and mutant locus could be identified according to the profile of difference. We enumerate all the possible profiles of difference and infer the mutant proportion according to Eq.(4). Subsequently, mutant locus and mutant base can be inferred easily. At last, according to Eq.(7), we calculate the score *S_p_*. For a given profile of difference, the calculation procedure is presented in Fig. 3.


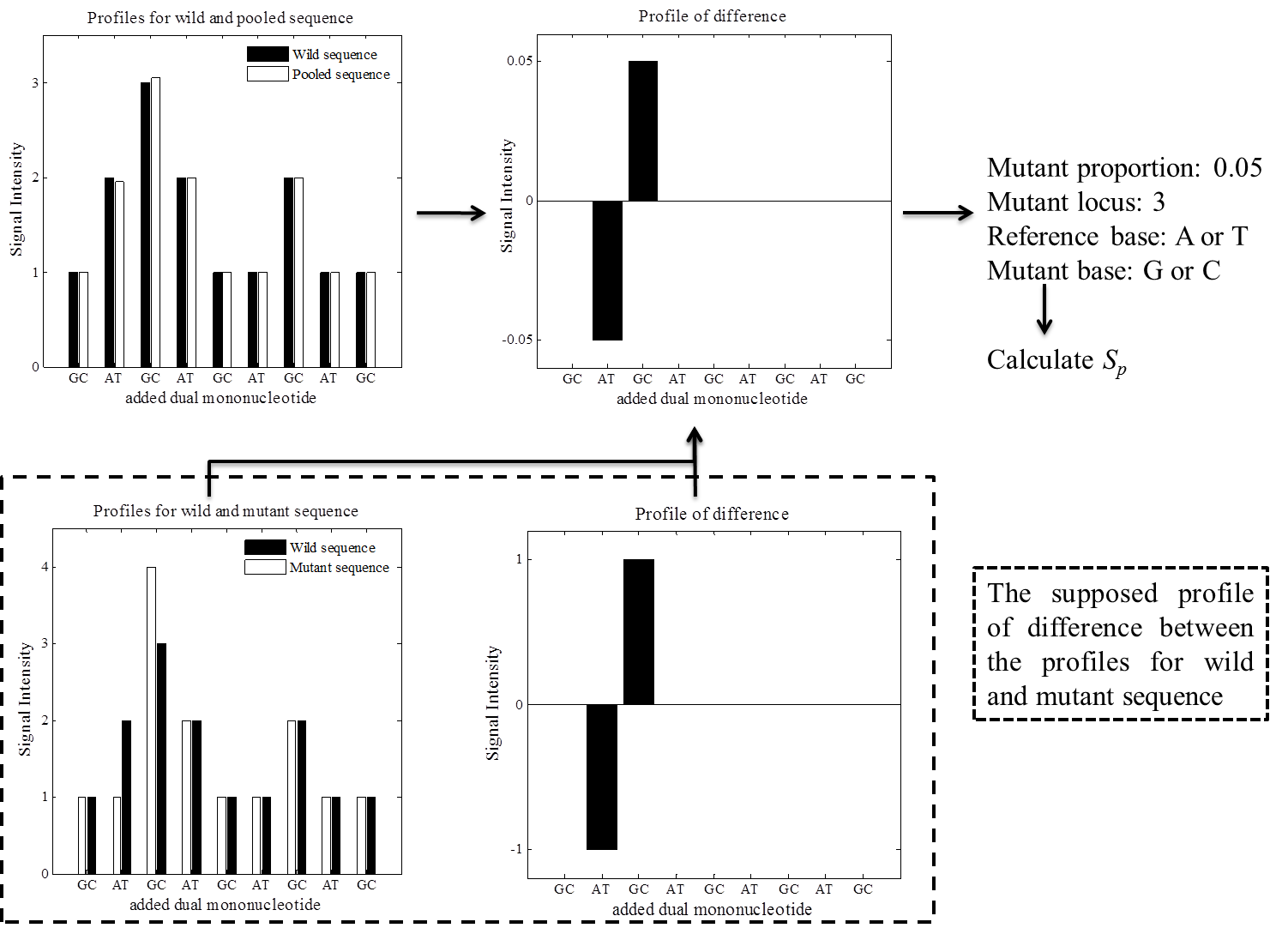


Fig.3 Estimate mutant proportion and infer mutant locus and mutant base according to the difference between the profiles of wild and pooled sequences.

4. Mutant recovery. Given pyrogram profiles resulting from three runs, we can infer mutant proportion, mutant locus and mutant base for each pair of profiles for wild and pooled sequences. According to the value of *S_m_*, we can infer the run which produces identical profiles for wild sequence and pooled samples. Based on the profiles resulted from the other runs, we could obtain two inferred mutant loci and two estimated proportions. When these two loci are identical, the mutant locus can be determined directly and the final estimation for the proportion of mutant could be calculated as the average of the two estimated proportions. At last, we can recover the mutant bases (Fig. 4).


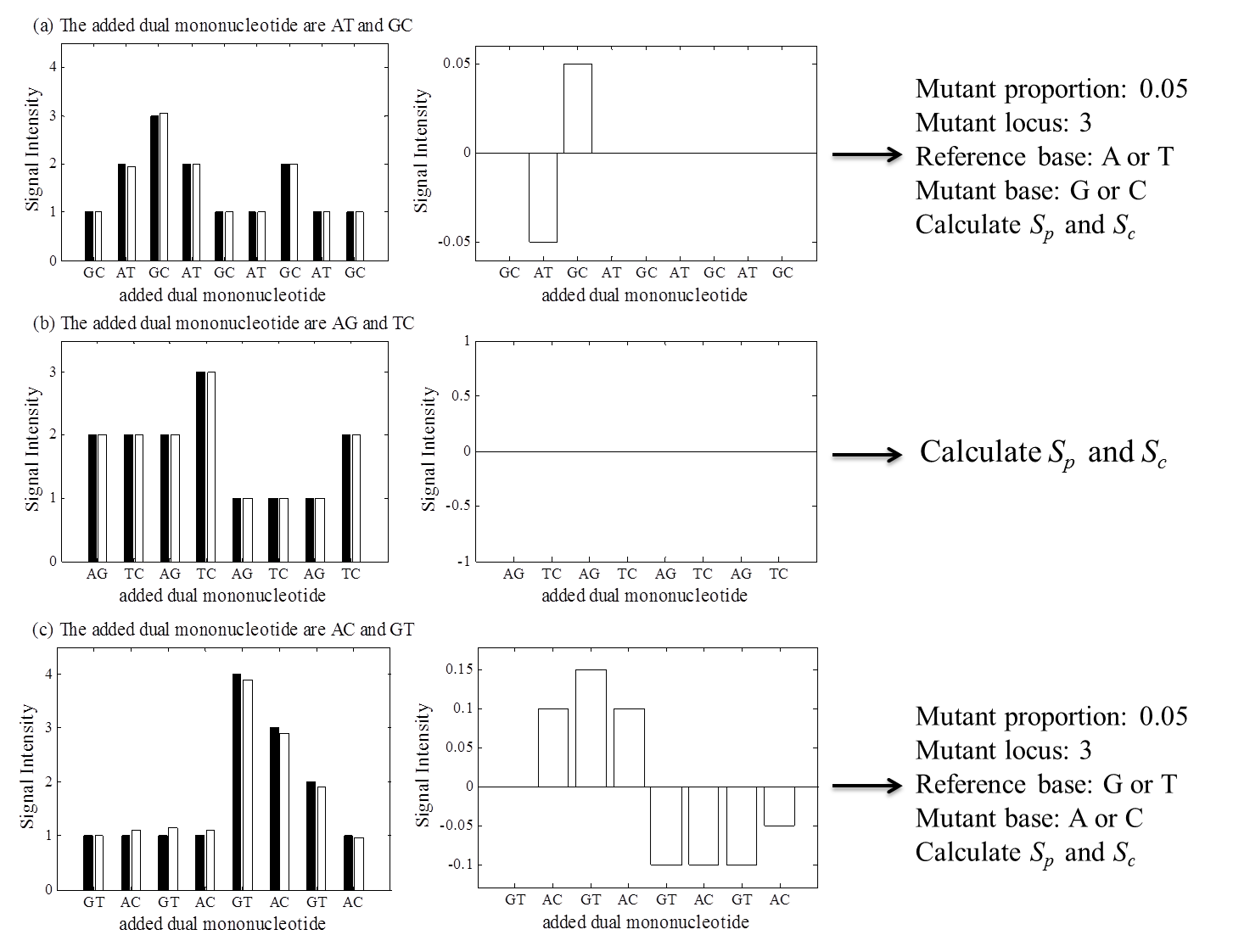


Fig.4 With three kinds of dual mononucleotide additions, the mutant in the pooled sample could be recovered correctly. (a) The added two kinds of dual mononucleotide are AT and GC. (b) The added two kinds of dual mononucleotide are AG and TC. (c) The added two kinds of dual mononucleotide are AC and GT. In this example, we can conclude that the mutant base is C, the mutant locus is 3 and the mutant proportion is 0.05.

**Appendix 2** Details about the protocol for sample preparation and pyrosequencing based on dual mononucleotide addition

1. Reagents

Streptavidin-coated Sepharose^TM^ High Performance beads were purchased from GE Healthcare (Uppsala, Sweden). PyroMark Q24 Advanced Reagents were purchased from Qiagen (Hilden, Germany). Details were listed as follows: PyroMark Q24 Advanced Kit (4×24, cat. no.970902), PyroMark Binding Buffer (cat. no.979006), PyroMark Denaturation Solution (cat. no.979007), PyroMark Wash Buffer (cat. no.979008) and PyroMark Annealing Buffer (cat. no.979009).

2. Genomic DNA extraction

Blood samples from 25 individuals were donated by the Campus Hospital of Southeast University (Nanjing, China). Genomic DNA was extracted from 200 μL whole human blood, using QIAamp DNA blood Mini Kit (Qiagen, Valencia, CA) following the manufacturer’s instructions. Qubit Fluorometer (Life Technologies, Carlsbad, USA) was used to determine the purity of genomic DNA.

3. PCR amplification

For SNP1 (rs4986850): the 153-bp target fragment of the *BRCA1* gene was amplified with the forward primer (5′-GGAAGGCTAGGATTGACAAATTCT-3′) and the reverse primer (5′-Biotin-TGGAGCCAAGAAGAGTAACAAGC-3′). For SNP2 (rs11564148): the 148-bp genomic fragment of the *LRRK2* gene was amplified with the forward primer (5′-AAGTGGAAGGTTGTCCAAAACACC-3′) and the reverse primer (5′-Biotin- CCTATTGGCAAAGCAATCTGGA-3′).1.5 ng of genomic DNA was used in a 50-μL reaction, containing 0.4 uM of the forward and reverse primers, Premix Taq™ (TaKaRa, Shanghai, China) and ddH_2_O. For SNP1, there was 35 thermal reaction cycling (98 °C for 10s, 48.8°C for 30s , 72°C for 30s) after initially denaturing at 94 °C for 3 min and a final extension at 72 °C for 5 min was set to ensure the complete extension of the amplified DNA fragment. For SNP2, the amplification was performed with the following protocol: initially denaturing at 94 °C for 3 min, followed by 35 thermal reaction cycling (94 °C for 30s, 60.2 °C for 30s, 72°C for 30s) and the identical final extension process for SNP1. Electrophoresis using 2% agarose gels was applied to verify PCR products and Sanger sequencing was used to determine concrete sequences.

4. Plasmid construction

Plasmids containing the wild or mutant allele for SNP1 and SNP2 were constructed by Invitrogen (Shanghai, China), utilizing the PCR products of genomic DNA. However, plasmid containing the mutant allele for SNP1 was not constructed successfully, maybe due to its low allele frequency and the limited sample size (blood sample from only 25 individuals). Subsequently, plasmid containing the mutant allele for SNP1 were generated by artificial synthesis, since both plasmid containing wild allele and plasmid containing mutant allele were required for simulating pooled samples containing mutant allele. Genotypes of all the plasmids were confirmed by Sanger sequencing. Next, plasmids were used as templates to obtain specific PCR products containing different proportions of mutant allele for SNP1 or SNP2. Pooled plasmid samples (P1-P4 for SNP1, P5-P8 for SNP2) consisting of wild and mutant sequences with different proportions were prepared respectively (Table 1).

Table 1. Pooled samples used in the verification experiment.

| Pooled plasmid samples | | Proportion of mutant type | Composition | |
| --- | --- | --- | --- | --- |
|  |  |  | Mutant type  (1.25 ng/ μL) | Wild type  (1.25 ng/ μL) |
| SNP1 | P1 | 0 % | 0 μL | 100 μL |
|  | P2 | 3% | 3 μL | 97 μL |
|  | P3 | 5 % | 5 μL | 95 μL |
|  | P4 | 7 % | 7 μL | 93 μL |
| SNP2 | P5 | 0 % | 0 μL | 100 μL |
|  | P6 | 3% | 3 μL | 97 μL |
|  | P7 | 5 % | 5 μL | 95 μL |
|  | P8 | 7 % | 7 μL | 93 μL |

5. Template DNA preparation

Pooled samples listed in Table 1 were obtained following the procedure of PCR amplification. Biotinylated DNA strands were immobilized on the Streptavidin-coated beads. The immobilization was performed as following: for each sample, the PCR product (10 μL) was transferred into a new PCR tube, then 40 μL binding buffer, 1 μL beads, 29 μL High purity water(18.2MΩ) were added and mixed at room temperature,1400 rpm for 15 min (Thermomixer Comfort, Eppendorf). Single-stranded DNA was obtained by using the standard protocol for the Vacuum Prep Tool (Biotage AB, Sweden). The single-stranded DNA was hybridized to 7.5 pmol sequencing primer (5′-CCAGGTGCATTTGTTAACTTCAG-3′ for SNP1, 5′-TGTGGAAAAATTTCTTTCAAA-3′ for SNP2) in 20 μL annealing buffer at 80°C for 5 min and then put into PyroMark Q24 Advanced instrument (Qiagen, Hilden, Germany) immediately.

6. Pyrosequencing with dual mononucleotide addition

There are six available dual mononucleotide combinations (AG, CT, AC, GT, AT, CG) with respect to the nucleotide addition, and all the four nucleotides (A, T, G, C) are essential for the sequences extensions in pyrosequencing, hence there are three kinds of sequencing combination for circulating addition: AG/TC, AT/CG and AG/CT. The pyrosequencing reactions were conducted using PyroMark Q24 Advanced reagents and PyroMark Q24 Advanced Instrument with the PyroMark Q24 Advanced software (Qiagen, Hilden, Germany). The Pyrograms were analyzed and the corresponding peak heights were obtained by using AQ mode of the PyroMark Q24 Advanced software.

7. Signal normalization

In our experiment, the peak signals were normalized by reference to the signal intensity for the reaction in which the number of incorporated nucleotides was 1 (Table 2). In reality, similar to the normal 454 sequencing technique, peak signals could be normalized by reference to the signals measured for the known key sequence included in each DNA sample [1]. Considering that the total number of extended nucleotides for wild and pooled sequence are equal, we used the ratio between the sum of the signal intensities for wild sequence and pooled sequence to normalize the signal intensities for pooled sequence, in order to further eliminate the impact of the difference between the concentration of wild and pooled sequences.

Table 2 The signal intensity for normalizing peak signals.

|  | Added dual mononucleotide | signal intensity used for normalization |
| --- | --- | --- |
| SNP1 | GC/AT | signal intensity for the first reaction |
|  | CT/AG | signal intensity for the third reaction |
|  | AC/GT | signal intensity for the first reaction |
| SNP2 | GC/AT | signal intensity for the second reaction |
|  | CT/AG | signal intensity for the first reaction |
|  | AC/GT | signal intensity for the second reaction |

Reference

1. Margulies M, Egholm M, Altman WE, Attiya S, Bader JS, Bemben LA, Berka J, Braverman MS, Chen Y-J, Chen Z: **Genome sequencing in microfabricated high-density picolitre reactors**. *Nature* 2005, **437**(7057):376-380.
